# Behavioral variation across the days and lives of honey bees

**DOI:** 10.1016/j.isci.2022.104842

**Published:** 2022-08-08

**Authors:** Michael L. Smith, Jacob D. Davidson, Benjamin Wild, David M. Dormagen, Tim Landgraf, Iain D. Couzin

**Affiliations:** 1 Department of Collective Behaviour, Max Planck Institute of Animal Behavior, 78464 Konstanz, Germany; 2 Department of Biology, University of Konstanz, 78464 Konstanz, Germany; 3 Centre for the Advanced Study of Collective Behaviour, University of Konstanz, 78464 Konstanz, Germany; 4 Department of Biological Sciences, Auburn University, Auburn AL 36849, USA; 5 Department of Mathematics and Computer Science, Freie Universität Berlin, 14195 Berlin, Germany

**Keywords:** Wildlife behavior, Ethology, Methodology in biological sciences

## Abstract

In honey bee colonies, workers generally change tasks with age (from brood care, to nest work, to foraging). While these trends are well established, our understanding of how individuals distribute tasks during a day, and how individuals differ in their lifetime behavioral trajectories, is limited. Here, we use automated tracking to obtain long-term data on 4,100+ bees tracked continuously at 3 Hz, across an entire summer, and use behavioral metrics to compare behavior at different timescales. Considering single days, we describe how bees differ in space use, detection, and movement. Analyzing the behavior exhibited across their entire lives, we find consistent inter-individual differences in the movement characteristics of individuals. Bees also differ in how quickly they transition through behavioral space to ultimately become foragers, with fast-transitioning bees living the shortest lives. Our analysis framework provides a quantitative approach to describe individual behavioral variation within a colony from single days to entire lifetimes.

## Highlights

• Automated barcode tracking follows the lifetime behavior of thousands of bees
• Quantitative analysis framework reveals considerable variation among age-matched bees
• Individual bees exhibit consistent differences in movement across their lifetimes
• Bees differ in when they transition their behavioral repertoires to become foragers

## Graphical abstract

**Figure:**
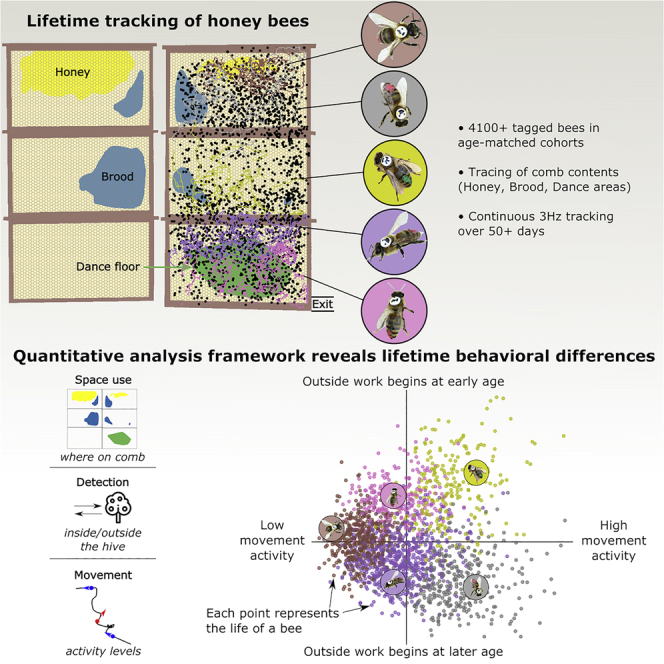

## Introduction

Social insect colonies are comprised of individual organisms that form a cooperative entity to propagate their genes (Seeley, 1989; Wilson and Sober, 1989; Smith and Szathmary, 1995). To survive, grow, and reproduce, a colony must navigate the same biotic and abiotic challenges as unicellular and multicellular organisms, but coordination must now occur at the level of individual workers (Hölldobler and Wilson, 2009). Social insect colonies lack centralized control, but across the ants, bees, termites, and wasps, tasks are instead self-organized among workers, whether genetically, physiologically, spatially, or temporally (Oster and Wilson, 1978; Seeley, 1982; Porter and Tschinkel, 1985; Jeanne, 1986; Jeanne et al., 1988; Frumhoff and Baker, 1988; Robinson et al., 1989, 2009; Fewell and Page, 1993; O’Donnell and Jeanne, 1995; Gordon, 1996; Naug and Gadagkar, 1998; Beshers and Fewell, 2001; Oldroyd and Fewell, 2007; Jandt and Dornhaus, 2009; Mersch et al., 2013; Baudier et al., 2020). Understanding how individuals combine to form a collective provides insights into the evolutionary drivers of organization across biological scales (Smith and Szathmary, 1995; Davidson et al., 2021).

A key challenge for highly integrated collective systems, such as eusocial insects, is how to allocate tasks among the individual units. While a fixed allocation strategy may be efficient in stable environments, a flexible approach allows colonies to respond to changing conditions (Gordon, 2014, 2016). Responsive (and decentralized) changes in task allocation can arise, for example, from individuals with different response thresholds for task-specific stimuli (Bonabeau et al., 1997), individuals selecting tasks based on current need or availability (Tofts, 1993; Jeanne, 1996), state-dependent probabilities to switch or remain in a current task (Gordon, 1999; Goldsby et al., 2012), age, developmental, or physiological task engagement (Seeley, 1982; Robinson et al., 1989; O’Donnell and Jeanne, 1993; Cook et al., 2019), or a combination of these mechanisms (Johnson, 2010). These mechanisms can also depend on the type of task: non-specialized tasks may be distributed widely among colony members, whereas tasks requiring certain physiological abilities may be restricted to specific individuals (Johnson, 2003; Robinson et al., 2009). Across social insect species, how and when tasks are allocated among individuals represents a balance between robustness and flexibility in colony function (Charbonneau and Dornhaus, 2015).

In colonies of the Western honey bee *Apis mellifera* individuals perform different tasks according to multiple factors, including developmental state, genetics, and behavioral feedback mediated by social interactions (Huang et al., 1994; Beshers and Fewell, 2001; Robinson, 2002; Grozinger et al., 2007; Johnson, 2008b; Cook and Breed, 2013; Cook et al., 2019; Wild et al., 2021). This gives rise to a general tendency for young bees to care for brood in the center of the nest, middle-age bees to perform various tasks throughout the nest, and old bees to forage outside and advertise food sites with waggle dances on the dance floor (Seeley, 1982). Within these general trends, individuals may switch between tasks, or perform multiple different tasks in a day; therefore, individual behavior is better described with “task-repertoires” — groups of tasks that are similar behaviorally and/or spatially (Seeley, 1982; Johnson, 2010). Although task repertoires vary with age, an age-based categorization does not account for variation among individuals throughout their lives, or how previous social and/or environmental experiences may influence task allocation (Jeanson and Weidenmüller, 2014; Beshers and Fewell, 2001; Wild et al., 2021).

While previous studies have relied on human observation to assign behavior to individuals using ethograms (e.g. Lindauer (1952); Seeley (1982); Seeley and Kolmes (1991); Johnson (2003); Siegel et al. (2013); Smith et al. (2017); Perez and Johnson (2019)), recent advances in automated tracking make it possible to extract behavioral metrics beyond the scope and scale of human observation (e.g. continuous location and instantaneous speed) (Mersch et al., 2013; Crall et al., 2015, 2018; Wario et al., 2015; Wild et al., 2018; Gernat et al., 2018; Jones et al., 2020; Richardson et al., 2021; Bozek et al., 2021). This allows one to move from general trends to detailed, long-term, quantification of behavior. The use of quantitative metrics to characterize behavior enables a data-driven approach to investigate the causes and consequences of individual variability and inter-individual differences across timescales.

In this study, we present data and analyze the behavior of 4,100+ honey bees across 16 age-matched cohorts tracked within an observation hive for 50 + days throughout a summer (July–October 2018). We define an analysis framework using behavioral metrics calculated from the motion data that quantify bees’ space use, detection, and movement. We use this framework to examine behavioral variation among age-matched bees, as well as variation in the behavioral trajectories of individuals over lifetimes. This analysis framework enables a quantitative comparison of the behavior of thousands of individuals at different timescales.

## Results

### Long-term tracking of individually marked bees

**Figure 1:**
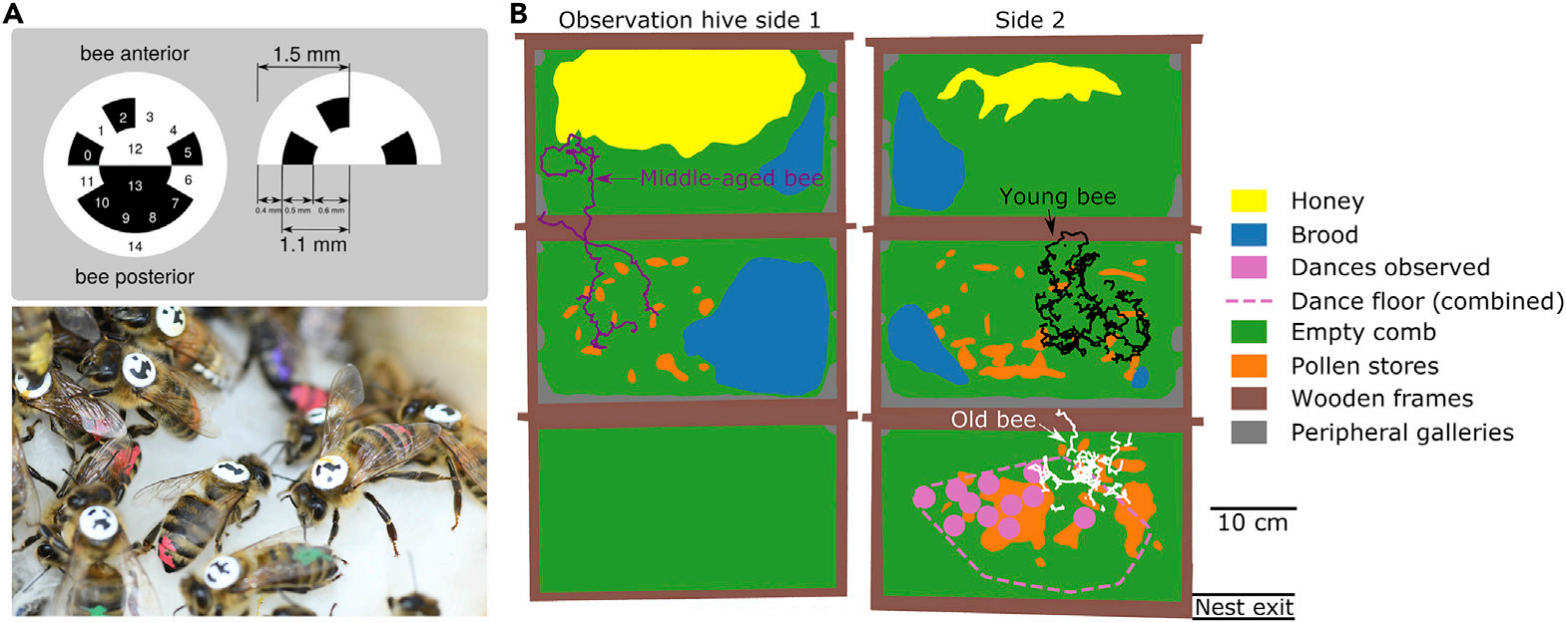
Long-term honey bee tracking (A) Bees were individually marked with barcodes, and tracked using the BeesBook tracking system (Boenisch et al., 2018). (B) An example map of the observation hive, with colors to denote different nest substrates. Dots overlaid on the map show trajectories of three representative bees with short trajectories selected from 11 August 2018: (black) young bee, age 6 days; (purple) middle-aged bee, age 16 days; (white) old bee, age 26 days. Nest exit/entrance at the lower right corner.

We tagged and tracked over 4,100 individuals, 3 times per second (3 Hz), day and night for 50 + days during summer 2018 using the using the BeesBook tracking system (Boenisch et al., 2018) (Figure 1A). Newborns were introduced to the 3-frame observation hive every 4–6 days, in cohorts of 200–600 bees. Each time a new cohort was introduced, we recorded the comb contents in the observation hive (as in Smith et al., 2016) to map the honey stores, brood nest, and dance floor. The dance floor is an area typically near the next exit, where foragers advertise food sites with waggle dances (Seeley, 1995). These content maps allow us to determine the context of the spatiotemporal patterns of activity exhibited by bees throughout their lives, in the context of their changing social and structural nest environment (Figures 1B and S1).

To quantify the activity of individual bees on a given day, we compute multiple behavioral metrics, which describe space use (time on honey, brood areas, and dance floor, and median exit distance), detection (time observed, time outside, number of outside trips, and number of dance floor visits), and movement (median speed, speed circadian coefficient, dispersion, and fraction of the nest visited). See Figure 3 for a visual depiction of these metrics, and see STAR Methods and Table 1 for a complete description of how each is computed.

**Table 1:** Behavioral metrics used in the analysis

Metric	Definition and description
Honey, Brood, Dance floor	Fraction of observed time spent on these substrates, as defined using the comb maps of Figure S1. For days when the comb was not measured, we used a weighted average with the closest measurement days.
Exit distance	Median shortest path distance to the exit (which is located in the lower right corner), accounting for possible routes to switch sides, but not adding any extra distance for a switch of sides.
Time observed	Total time observed in a day, calculated as the total number of detections with confidence interval over the 0.8 detection threshold, divided by the frame rate of 3 frames per second.
Time outside	An estimate of the total amount of the time a bee spends outside during a day.
Number of outside trips	An estimate of the number of times a bee exited the nest in a day.
Number of dance floor visits	The number of times that a bee entered the dance floor from another substrate.
Median speed	Median speed during time observed, omitting instances where the bee switched sides of the comb, as well as when the time between detections was >1 second.
Circadian coefficient	A representation of how activity levels change with the time of day; positive values represent higher observed speeds during the day, while negative values represent higher speeds at night.
Dispersion	Root mean square distance from the centroid of the x-y coordinates, calculated by considering motion in a 2D plane in the hive (i.e. neglecting whether the bee was detected on the front or the back of the observation hive).
Fraction nest visited	After dividing the nest area into discrete spatial bins of 2 cm × 2 cm (the same grid size used in the spatial histograms shown in Figure 4), this is the fraction of bins with at least one detection. Note that the body size of a bee is approx. 1 cm.

See STAR Methods for precise descriptions of how substrate usage (honey, brood, and dance floor), circadian coefficient, and time outside/outside trips are calculated.

**Figure 2:**
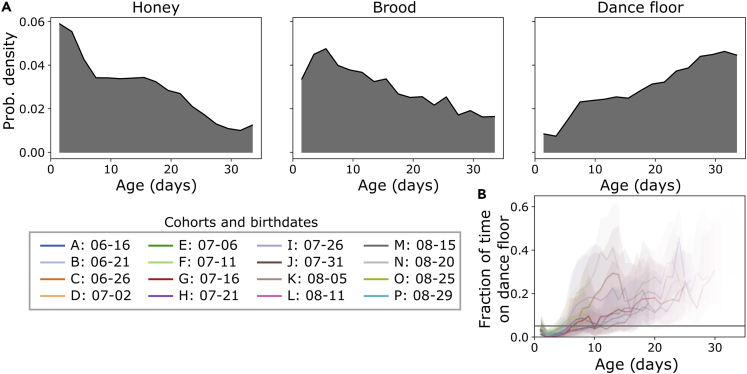
Bee nest usage histograms and changes with age A substrate usage histograms with respect to age. (B) Cohort distributions of dance floor usage with age. Colors represent the different cohorts, ordered chronologically by birthday, with corresponding alphabetical names. Lines show the mean and the shaded area shows the standard deviation across bees in each cohort. The transparency is proportional to the fraction of bees in a cohort that lived to a certain age. See also Figure S2.

At any given time, bees on honey storage and brood areas tend to be younger than bees on the dance floor (Figure 2A). This trend is consistent with the well-established sequence of young workers performing within-nest tasks, and old workers foraging outside (Seeley, 1982; Robinson, 1992). As individuals age, they spend more time on the dance floor, but we observe considerable differences among bees within the same age-matched cohort on any given day (Figures 2B and S2).

### Individual behavior during a single day

**Figure 3:**
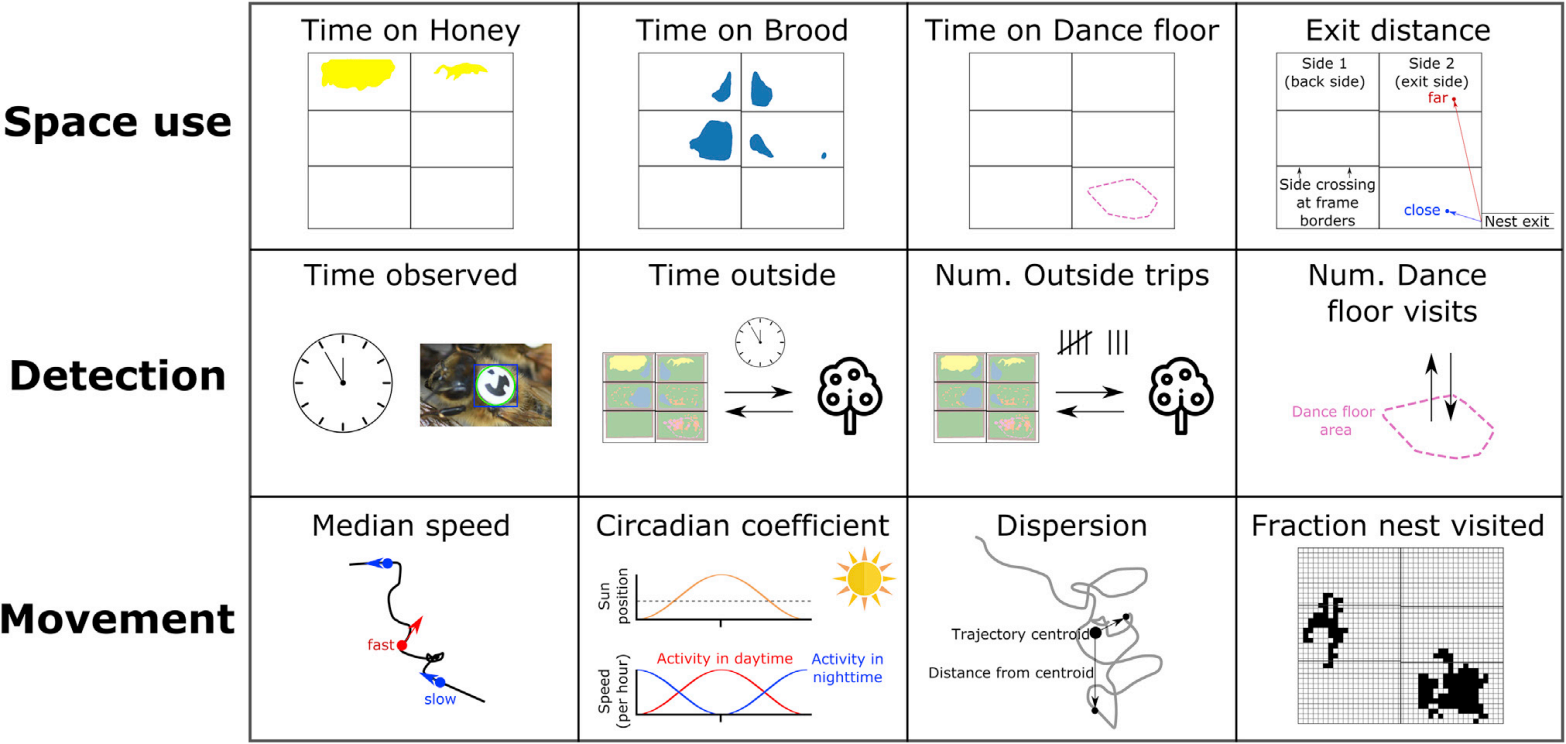
Metrics used to quantify behavior of tracked bees We use 12 behavioral metrics to quantify the behavioral of individual bees. These are grouped in metrics describing space use (time spent on honey, brood, and dance floor areas, and median exit distance), detection (time observed, time spent outside the nest, number of outside trips, and number of dance floor visits), and movement (median speed, speed circadian coefficient, dispersion, and fraction of the nest visited). See Table 1 and STAR Methods for further details on how each metric is computed.

In this section, we examine single-day behavioral variation. We use the term “behavioral day” to refer to the behavior of a single bee on a single day. Quantitatively, a behavioral day refers to the behavioral metrics shown in Figure 3 calculated on a single day for a single bee. Note that individual bees have multiple “behavioral days” that make up their life, and may exhibit different behavior on different days—in this section, we describe differences in individual behavioral days irrespective of individual identity, and then in the next section we use the known identity of each bee to compare how individuals change their behavior over time. For consistency in comparing behavior, we focus the analysis on the 50-day period during which new cohorts were added every 4–6 days. In this time period, the dataset includes a total of 53,032 behavioral days, which are from 4,193 tracked bees.

We use principal component analysis (PCA), clustering, and visualization methods to describe the space of behavioral variation (Valletta et al., 2017). Note that this does not assign specific activities (e.g. fanning) to individuals over time, like an ethogram, but instead uses behavioral metrics computed from the barcode-tracking data to identify patterns and similarities in behavior among behavioral days. The behavioral metrics include time on honey, brood, and dance floor areas, exit distance, time observed, time outside, number of outside trips, number of dance floor visits, median speed, speed circadian coefficient, dispersion, and fraction of the nest visited; these 12 metrics represent space use, detection, and movement and are graphically depicted in Figure 3 and defined in full detail in Table 1.

PCA extracts the dominant axes of behavioral variation, i.e. the relative weightings of the behavioral metrics that explain the largest percentage of variance in the data matrix. To perform PCA, we first arrange the data in a matrix structure, normalize each metric so that all can be compared in the same standardized units, and then calculate the PCA decomposition. In the day-data matrix $M_{ij}$, each row i=1…53,032 is for a single behavioral day (i.e. a single tracked bee on a single day), and the columns j=1…12 are for each behavioral metric. The data matrix is normalized following standard procedures by subtracting the mean and dividing by the standard deviation of each column. With this, the total variance is simply the matrix norm and is equal to the number of metrics, i.e. $\|M_{ij}\|=12$, and the percentage of variance explained can be computed using the remaining variance after subtracting a particular pattern from the data matrix (Jolliffe, 2002; Valletta et al., 2017). Because of the normalization, positive/negative weightings in the PCA components represent higher/lower values of a metric with respect to the average across all behavioral days.

**Figure 4:**
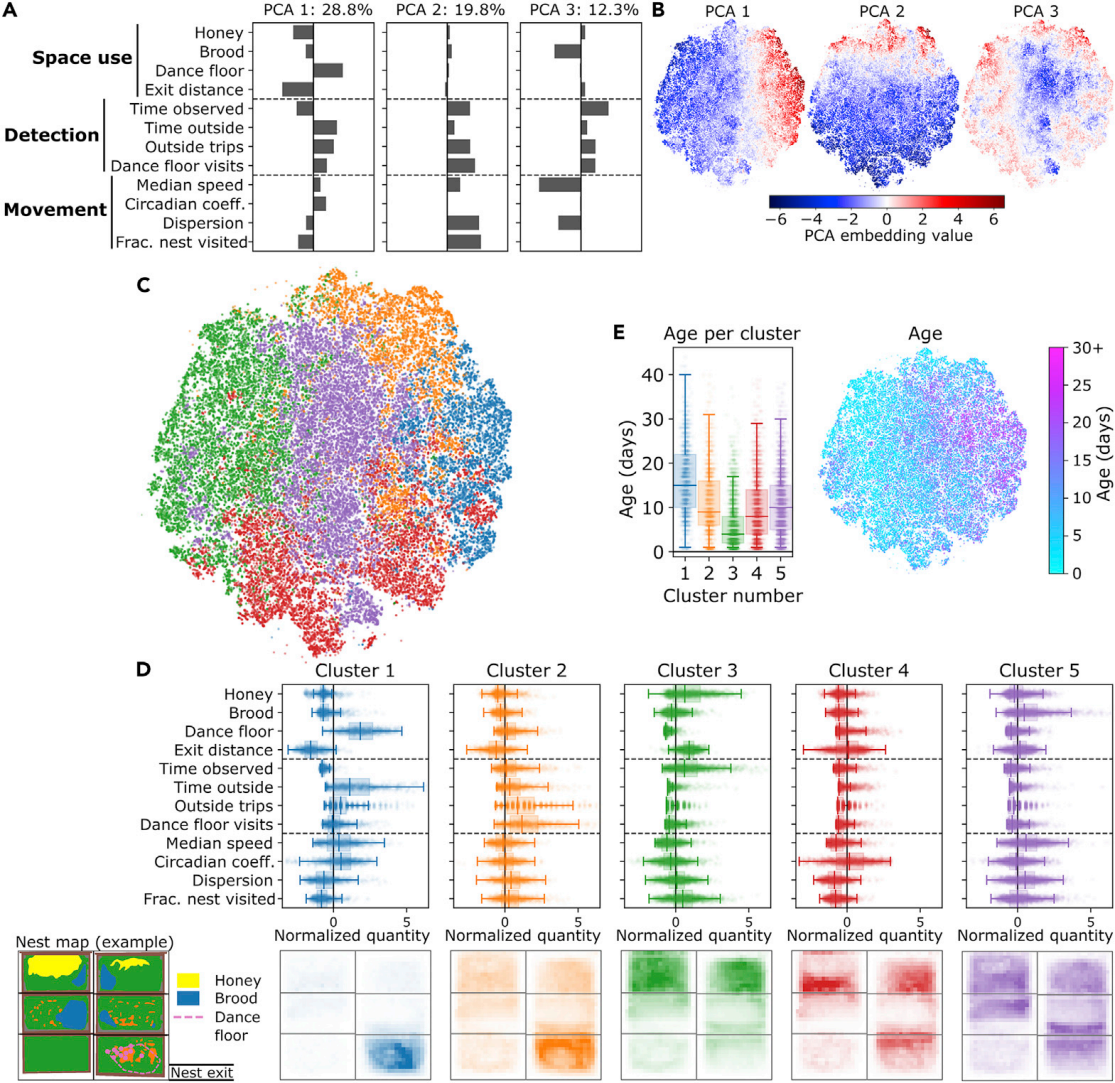
Differences in observed single-day behavior (A) The first three components from the PCA decomposition of individual bee behavioral metrics on a given day. (B) t-SNE embedding of behavioral days, colored by the projection values along each PCA component dimension. The t-SNE is initialized with the first two PCA component projections, and therefore the global structure of the t-SNE embeddings aligns with these projections. (C-D) Distributions of behavioral days using 5 clusters, showing (C) these groups plotted with different colors on the t-SNE embedding and (D) behavioral metrics and average nest location histograms. Nest histograms reflect the layout of the observation hive, sample shown at the bottom right for reference, and in Figures 1B and S1. (E) Age distributions of each behavioral day cluster. See also Figure S3.

We find that the first 3 principal components represent important axes of variation among behavioral days. The first PCA component explains the largest amount of variance (28.8%), and is strongly weighted by space use: in particular, time on the dance floor and low exit distance (Figure 4A). The second PCA component accounts for 19.8% of the variance and is strongly weighted by fraction of the nest visited and dispersion, which are two complementary metrics that represents how wide-ranging a bee is, regardless of where it tends to be located in the nest (see Figure 3 and Table 1 for descriptions of how these metrics are defined). The third PCA component (12.3% of the total variance) is most strongly weighted by speed and time spent on brood.

The first 3 components explain 60.9% of the total variance of all behavioral metrics included in the normalized data matrix $\left(M_{ij}\right)$, with the first component accounting for 28.8%. In comparison, the total amount of variance explained by age alone is only 9.2%, and that by age & cohort is only 17% (see Table 2). This demonstrates that although there are indeed consistent trends among bees as they age, as well as differences across cohorts, age and cohort alone are insufficient to account for the full range of behavioral variation in the data. We therefore use a more general method to describe the dominant daily behavioral differences among bees.

**Table 2:** Amount of single-day behavioral variation explained by cohort and age

Metric	Variance explained by grouping (percentage)		
	Age&Cohort	Age	Cohort
Honey	11.0	3.9	5.8
Brood	7.7	2.9	1.7
Dance floor	26.6	19.3	2.7
Exit distance	28.7	23.1	2.6
Time observed	27.1	17.1	14.2
Time outside	15.3	10.6	2.1
Num. outside trips	12.6	5.9	2.1
Num. dance floor visits	12.7	2.7	4.6
Median speed	27.0	14.4	6.8
Speed circadian coeff.	10.4	6.1	2.4
Dispersion	9.9	0.6	6.8
Fraction nest visited	14.5	3.7	10.1
All data and metrics	17.0	9.2	5.2

This is calculated by subtracting the conditional average of each metric from the normalized behavioral day-data matrix, where the conditional averages are calculated using groupings of age&cohort, age, or cohort. Values are shown for each metric using Equation 3, as well as all metrics combined (Equation 2).

To visualize the multi-dimensional range of variation in the data matrix $\left(M_{ij}\right)$, we use t-distributed stochastic neighbor embedding (t-SNE; Maaten and Hinton 2008). Each “data point” in this embedding is a single behavioral day. Because it is initialized with the first two PCA components, the global structure of the t-SNE embedding corresponds to the first two PCA components, but the local structure can represent higher-order PCA components (Figure 4B). This embedding is therefore a useful visual aid to display the range of single-day behavioral variation in a compact yet meaningful way.

We apply Ward hierarchical clustering (Ward Jr, 1963; Valletta et al., 2017) to group behavioral days with similar behavioral metrics. Examining the dendogram structure and the within-cluster variance as a function of number of clusters, we find that distinct behavioral clusters do not exist in our data (Figure S3) However, with this clearly in mind, we can still employ clustering tools to aid in the comparison, visualization, and thus the understanding, of the trends we see in the behavioral metrics of bees on different days (Figure 4C-D). To this end, we focus on the 5-cluster result as a practically useful grouping to describe dominant trends in the data, as represented by differences in the first 3 PCA components.

Each data point in Figure 4C represents the behavioral metrics calculated for a single bee on a single day; a “behavioral day” (see Figure 3 for behavioral metrics). Cluster 1 (blue) represents behavioral days that have a high dance floor use and time outside, with higher activity during the day (higher than average speed circadian coefficient), and the highest median age. Similar to cluster 1, cluster 2 (orange) represents behavioral days in which bees spent time close to the exit; however, in contrast to cluster 1, cluster 2 behavioral days have particularly high number of outside trips and dance floor visits, with visits to multiple areas of the nest, not just the dance floor and exit frame—see histograms in Figure 4D. Cluster 3 (green) represents behavioral days in which bees have the highest distance to the exit and time spent on honey areas, in comparison with other clusters. Cluster 4 (red) represents behavioral days associated with middle-aged bees, with metrics representing slow, localized behavior, mostly on empty-comb “border” regions of the nest (hence the lower than average time on both honey and brood areas). Cluster 5 (purple) represents behavioral days with higher than average speed and dispersion, as well as a higher than average time on brood areas.

These groupings illustrate the dominant differences among behavioral days. While cluster 1 and cluster 2 behavioral days represent similar space use, they have differences in detection and movement; both represent behavioral days where bees exited the nest, but cluster 1 represents more time outside, while cluster 2 represents more trips and visits to places in the nest other than just the dance floor. Clusters 3, 4, and 5 are similar in detection (these behavioral days are mostly inside the nest), but differ in space use and movement. Cluster 3 behavioral days are farthest from the exit and have the highest average time on honey areas, while cluster 4 behavioral days have high time spent on areas that border brood and honey, and cluster 5 has high time spent on brood areas. Cluster 3 and 4 behavioral days have lower movement speeds, while cluster 5 has higher speeds. Although there is much overlap in the age distributions, cluster 3 behavioral days have the lowest median age (4 days), clusters 2, 4, and 5 have intermediate values (median ages of 9, 8, and 10 days, respectively), and cluster 1 the highest median age (15 days) (Figure 4E).

### Behavioral variation over lifetimes

In this section, we use individual identities to compare lifetime behavioral trajectories among individual bees. Bees are known to change their behavior over time due to internal processes such as physiological development, interactions with other bees, and environmental factors (Robinson, 1992; Amdam and Omholt, 2003; Johnson, 2003, 2010; Wild et al., 2021). To quantitatively compare how different bees change behavior as they age, we use a procedure similar to that used for behavioral days. However, now instead of a single day, each data point represents the entire life of an individual bee — we refer to this as a “bee-life”. Quantitatively, a bee-life is defined by the behavioral metrics for each day of the bee’s life (i.e. the series of behavioral days that make up an individual’s life).

**Figure 5:**
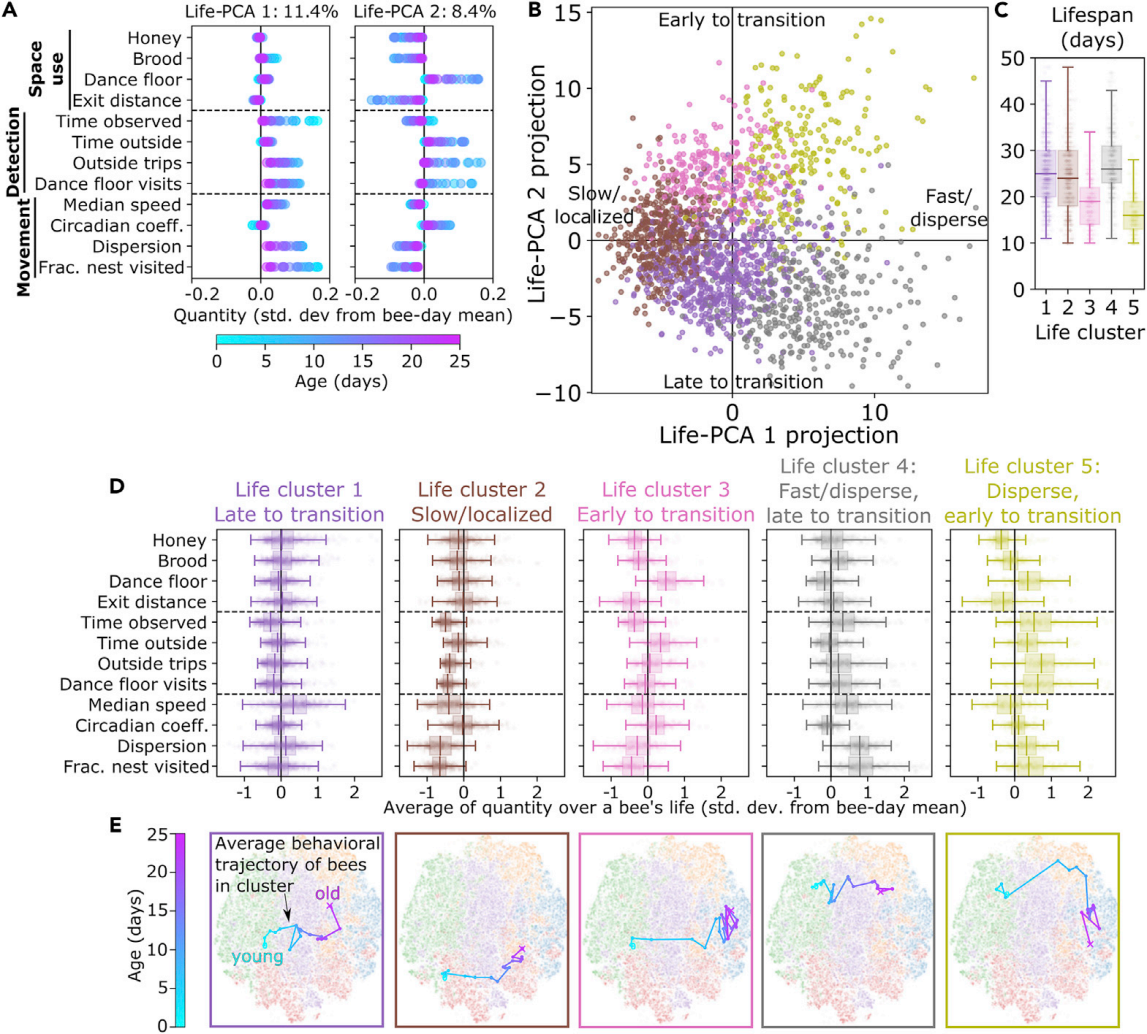
Behavioral differences across a bee’s entire life (A) PCA decomposition of bee-lives shows the dominant modes for how the behavioral metrics change over time. Plots show each PCA mode plotted in terms of behavioral metrics, using normalized quantities with the same units as in Figure 4 (i.e. zero represents the mean of the behavioral metric across all behavioral days). Points are colored by the age corresponding to each metric (see Figure S4A for an expanded plot). (B) A plot of individual bee-lives projected onto the first two PCA modes. Each point represents the life of a single bee. The colors correspond to a 5-cluster division, identified via Ward hierarchical clustering, and the labels describe the different life-PCA axes. (C) Distributions of number of days lived for bees in each life cluster. Note that bees are only included if they lived at least 10 days. (D) The distribution of the lifetime average of each behavioral metric among individual bees, grouped by bee-life cluster. The x axis is the average of each quantity during a bee’s life, in units of standard deviations from the mean of all behavioral days. (E) The average lifetime behavioral “trajectory” of behavioral days in each life cluster, projected onto the behavioral day embedding space. This is determined by averaging the metrics of bees of a certain age in each cluster and projecting these averages onto the behavioral day t-SNE embedding shown in Figures 4C, (Poličar et al. (2019); see STAR Methods). Points and connecting lines are colored by age. See also Figure S4.

We again use PCA and clustering to describe behavioral variation among bee-lives. To do this, we arrange the data into a three-dimensional matrix form to represent individual behavioral metrics for each day of a bee’s life. The bee-life data matrix is of the form $B_{\alpha tj}$, where α is for individual bees, t is an index over the days of the bee’s life, and j is for the different behavioral metrics (these are the same as used in the per-day analysis; see Figure 3). The life-PCA decomposition considers each bee α as a single input entry; the components can thus represent both consistent lifetime differences in behavioral metrics, as well as changes in behavior over time (Figures 5A and S4A). Note that due to the high-dimensional input (i.e. all behavioral metrics over multiple days), each PCA component in the lifetime analysis represents a comparatively smaller fraction of the variance, as compared to the per-day analysis shown previously in Figure 4. The first two life-PCA components nonetheless represent strong trends in the data (Figure S4B), and we focus our interpretations on these first two life-PCA components.

The first life-PCA component predominantly represents an overall difference in movement activity across the whole lifetime of a bee (Figures 5A and S4A). Positive projections of lifetime data onto the first life-PCA component represent bees with higher movement activity (higher dispersion and fraction of nest visited), and negative projections represent lower movement activity than average. The second life-PCA component represents differences in space use—both averaged across a lifetime, as well as changes with age (Figures 5A and S4A). Positive projections of lifetime data onto the second life-PCA component represent an early transition to increased dance floor use and time outside, while negative projections represent a lesser or later increase in these metrics.

Applying hierarchical clusterical to group bee-lives with similar behavior, the dendogram structure (Figure S4C) and the within-cluster variance (Figure S4D) suggest a continuous range of variation in lifetime behavior. As with the per-day results, we therefore use clustering as a descriptive tool. We use a 5-cluster grouping to describe, compare, and visualize differences in the lifetime behavior of bees along the dominant two life-PCA components (Figure S4E). To visualize these groups, we show each bee as a single data point projected on the first two life-PCA components (Figure 5B), the lifespan of bees in each cluster (Figure 5C), the distribution of values of behavioral metrics averaged over the lifetime of bees in each cluster (Figure 5D), and the average behavioral changes with time as projected onto the behavioral day embedding (Figure 5E). Life cluster 1 includes bees with average movement activity levels, but with late transitions to dance floor/outside activity. Conversely, life cluster 3 bees are similar in their average movement activity (i.e. a similar projection onto life-PCA 1 compared to cluster 1 bees), but show an earlier transition to dance floor/outside activity. The average projection on the behavioral day embedding further demonstrates this trend: while bees in clusters 1 and 3 on average move through similar regions of behavioral space (both move “left-to-right”, going roughly through the middle of the behavioral day embedding space), life cluster 3 bees change their behavior at younger ages than life cluster 1 bees (Figure 5E).

A similar comparison differentiates life cluster 4 and 5 bees; although bees in both of these clusters have higher than average movement activity over their lifetimes (i.e. positive projections onto life-PCA 1), life cluster 4 bees transitioned at older ages to dance floor/outside activity in comparison with life cluster 4 bees. Life cluster 2 bees have the lowest movement activity (negative projection onto life-PCA 1), and average transition ages. Bees in life clusters 3 and 5—i.e. those that transitioned to dance floor/outside activities at younger ages—had shorter lifespans than bees in the other life clusters (Figure 5C).

While there is no correlation between the two PCA projections (this is true by definition of the PCA calculation), the points representing the individual bees are not uniformly distributed on the PCA axes (Figure 5B). High-movement activity bees (life clusters 4&5; positive projection on life-PCA 1) have a higher variance in observated rates of transitioning, compared to slow/localized bees (life cluster 1; negative projection on life-PCA 1). This means that while overall movement characteristics are not correlated with when a bee transitions to outdoor-related activities, high-movement activity bees tend to be at the extremes of the distribution (i.e. the earliest or the latest to transition bees are fast). Slow/localized bees have average ages of behavioral transitions and a lower variance among individuals.

The results of the bee-life analysis show that individuals exhibit lifelong behavioral consistencies: those with positive projections onto life-PCA 1 (e.g. those in life cluster 4) tend to move faster and visit more areas of the nest over their entire lives, and individuals with negative projections onto life-PCA 1 (e.g. those in life cluster 2) tend to move more slowly and visit less areas of the nest over their entire lives (5D). We also find that individuals differ in the timing of their developmental transitions (Figure 5E): individuals with positive projections onto life-PCA 2 (e.g. those in life clusters 3 and 5) transition at younger ages to outside activities in comparison with individuals with negative projections onto life-PCA 2 (e.g. those in life clusters 1 and 4). Moreover, we see that the timing of behavioral transitions is related to longevity: individuals who transitioned to outside activities at earlier ages lived shorter lives (Figure 5C).

## Discussion

Using individual tracking data from 4,100+ honey bees, we calculated behavioral metrics from the motion data and defined an analysis framework to describe behavioral variation at different timescales. At the timescale of a single day, bees differed their space use, detections, and movement, as quantified by the behavioral metrics shown in Figure 3. Although some behavioral patterns are more associated with older bees (e.g. behavioral day cluster 1), and others with younger bees (e.g. behavioral day cluster 3), we see considerable overlap in the age distributions associated with different behavioral days (Figure 4). Looking at the entire lives of individuals, bees predominantly differed in their movement patterns (speed/dispersion; Life-PCA 1), and the age at which they transitioned to dance floor/outside activities (Life-PCA 2) (Figure 5). We found that across entire lifetimes, some individuals exhibit consistently different movement characteristics—in particular, consistently higher (or lower) dispersion across nest areas over their entire lives (Figure 5).

Behavioral differences among individuals may enable eusocial insect colonies to be flexible in response to changing conditions, yet robust to the maintenance of other colony functions (Jandt and Gordon, 2016; Garrison et al., 2018). Individual tracking of bumblebees has revealed consistent differences in movement activity (Jandt and Dornhaus, 2009; Crall et al., 2018)—in particular, in the overall spatial area occupied by an individual (i.e. dispersion). Other work has shown, for example, that bumblebees differ in thermoregulation response thresholds (Jandt and Dornhaus, 2014), ants show consistent differences in exploratory behavior (Maák et al., 2020), and honey bees differ in dance activity in response to the same food source (George and Brockmann, 2019). It is important to note that the colony response is an emergent outcome of the many individuals, where each individual also adjusts their behavior in response to the behavior of others (e.g. Ulrich et al. (2021)). In general, the distribution of individual behavioral traits within a eusocial insect colony is expected to affect colony function, because the colony is the reproductive unit that selection acts upon (Jeanson and Weidenmüller, 2014; Jandt and Gordon, 2016). However, the effect of inter-individual variation may depend on the specific function. For example, while the effect of inter-individual differences in response thresholds on overall bumblebee colony thermoregulation behavior is unclear (Jandt and Dornhaus, 2014), variation in body size among bumble bee workers in a colony has been linked to enhanced comb production (Holland et al., 2021), and other work with ants has demonstrated that the distribution of individual traits affects colony foraging behavior (Kolay et al., 2020). To understand the effects of inter-individual variation on colony performance, it is therefore important to consider both the specific colony function as well as the ecological context (Gordon, 2016; Davidson et al., 2021).

It is well known that there is a genetic basis for behavior in honey bees (Calderone and Page, 1988, Calderone and Page, 1991; Robinson et al., 1989; Page and Robinson, 1991; Fewell and Page, 1993; Junca et al., 2019; George et al., 2020), which likely also applies to lifetime behavior. The cohorts used in this study came from naturally mated colonies; each source colony has a different queen, and while some cohorts came from the same source colony (see Figure S5), workers in a given source colony also represent multiple different patrilines (queens mate with 12 ± 6 drones; (Tarpy et al., 2004)). To examine precisely the extent to which our results have a genetic basis, future work could compare behavior from single-drone inseminated queens, or use genomic sequencing to determine each worker’s patriline (Junca et al., 2019). Patriline diversity is important for colony-level function (Jones et al., 2004; Seeley and Tarpy, 2007; Mattila and Seeley, 2007; Mattila et al., 2012)); whether a diversity in “bee-lives” (i.e. differences in movement characteristics and behavioral transitioning ages; Figure 5) contributes to colony function is unknown.

In our analysis, we find that bees differ in both movement characteristics and the age at which they transition to spending time on the dance floor and outside of the nest (Figure 5 and S4). Previous work has noted how age is not the only factor that determines task allocation and behavioral transitions; social interactions, colony state, and environmental conditions also play a role (Beshers and Fewell, 2001; Johnson, 2010; Jeanson and Weidenmüller, 2014; Wild et al., 2021). While we see differences in space use with age, in our analysis of movement characteristics, we find that average speed tends to increase with age but dispersion does not (Figure S2). For example, while age explains 9.2% of the variance of all metrics together, age explains only 0.6% of the variance in dispersion. The amount explained by age is 14.4% for speed, and as much as 23.1% for median exit distance (see Table 2). We also note that precocious foraging, which is similar to the “early-to-transition” individuals that we observe, can be induced via hormone treatments (Robinson et al., 1989), infection (Woyciechowski and Moroń, 2009), colony demography (Huang and Robinson, 1996), or even pesticide exposure (Hesselbach et al., 2020), but here we see that such individuals exist even in unmanipulated colonies, similar to the study by Wild et al., 2021. In wasps, differences in the age at which individuals transition to different tasks have also been observed (Jeanne et al., 1988). Across cohorts, individuals from cohort N did tend to show more early-to-transition behavior than bees in other cohorts (Figure S5) but further experiments would be needed to show whether such differences are driven by genetic or environmental factors.

Our study uses a large observation hive (3-frames; 7,252 cm^2^ of surface area), which is larger and can house more bees than other studies using automated tracking of honey bees (e.g. Wild et al. (2020); Jones et al. (2020); Bozek et al. (2021); Wild et al. (2021). It is possible that nest size influences task allocation or transition rates; for example, workers in smaller colonies may transition between tasks more frequently (Jeanne, 1986; Dornhaus et al., 2012)). The observation hive was designed to mimic natural conditions and provide sufficient space for spatially separated comb-use areas (e.g. a dance floor that does not overlap with brood). Still, it is smaller than a natural nest (mature natural nests can have 13,369 ± 1174 cm2 of comb surface area; Smith et al. (2016)). We note that a systematic comparison of how nest structure influences behavior should consider not only size but also nest geometry (e.g. Pinter-Wollman (2015)).

Previous work has used ethograms to define categorical age-based labels such as nurses, middle-aged bees, and foragers (Lindauer, 1952; Seeley, 1982; Seeley and Kolmes, 1991; Johnson, 2008a,b, 2010). While such labels have the advantage of being easy to interpret, manually assigning behavioral tasks has multiple disadvantages, including: limited reproducibility (ethogram interpretations depend on the observer), behavioral descriptions must fit into pre-defined categories, and scaling issues (tracking multiple bees simultaneously, or over long time-periods, can be infeasible). Although automated tracking methods address these issues, simple trajectory data may not always be of direct biological or functional relevance (Krause et al., 2013). In the current study, for example, we incorporate maps of the nest structure to extract additional biological information for a given spatial positioning (e.g. the individual is located atop brood, versus on the dance floor). With honey bees, tasks are often location-specific, such that, for example, bees found on the brood area are typically doing brood care (Seeley, 1982). However, using location to infer task is an assumption, and some tasks, such as fanning, may not be location-specific. This is an inherent tradeoff with high-throughput methods like automated tracking. An important area for future work is to compare and relate the results of automated tracking methods, to approaches that use ethograms to manually assign behavior and task repertoires (e.g. cell cleaning, fanning, and waggle dances) (Lindauer, 1952; Seeley, 1982; Mattila et al., 2012; Smith et al., 2017; Perez and Johnson, 2019).

Recent work has combined barcode tracking with supervised machine learning methods to automatically identify specific behavioral events (Gernat et al., 2020; Jones et al., 2020). These approaches apply convolutional neural networks (CNNs) to video data to identify a specific behavior of interest (e.g. egg-laying), which can be associated with the known identities of tracked bees through the barcode positions. Gernat et al. (2020) trained their CNN to detect trophollaxis events, and Jones et al. (2020) to detect egg-laying events and when bees exited for outside trips. These are supervised methods which require training and specified behavior to identify, and thus have focused on a few types of behavioral events which could be reliably identified. Alternatively, recent work has combined general methods of pose estimation with barcode tracking and applied this to bumblebees (Smith et al., 2022); such pose estimation data could be used with unsupervised methods in order to identify complex behavioral patterns without training or a-priori specification (Berman et al., 2014; Graving and Couzin, 2020). In contrast to these approaches, which use smaller colonies and shorter tracking periods of 2–7 days (Gernat et al., 2020; Jones et al., 2020; Smith et al., 2022), in this study, we extract only trajectory data from barcode tracking, which enables the analysis of thousands of bees during their entire lifetimes in a timespan of several months. Future work can merge these approaches or choose the methods most appropriate to specific biological questions, by combining aspects of supervised identification of behavioral events, unsupervised behavioral classification from pose estimation, and behavioral metrics calculated from trajectory data.

Automated tracking makes it possible to obtain long-term datasets for thousands of individuals, making it possible to investigate individual variation at an unprecedented scale. Our long-term tracking results present a detailed picture of how individuals in a colony differ in their behavior from day-to-day and over entire lifetimes, and establish an analysis framework that can quantify these differences and how they may contribute to colony function.

### Limitations of the study

In this study, we analyzed the data of thousands of honey bees tracked using barcodes in an observation hive over an entire summer. While we examine variation among behavioral days and across the lifetimes of individual bees, we note that the metrics used to quantify behavior are restricted to quantities that can be calculated from the trajectory data (see Figure 3 for behavioral metrics). As such, these metrics do not directly represent biologically relevant behavioral patterns, such as foraging, cell cleaning, or fanning, that are typically identified with manual observation. Future work could examine how the behavioral metrics calculated from trajectory data are correlated with such manual assignments of behavior.

Although we examined the behavior of thousands of bees from multiple age-matched cohorts, our data are from a single observation hive over a single summer. Given that the colony had free access to forage outside, and that behavior can change with environmental factors, we can expect results to differ quantitatively from year-to-year. Nonetheless, we expect that observed qualitative trends would be similar for such a repeated experiment. Future work would be needed to test the repeatability and robustness of the observed trends, given the colony-level sample size.

## STAR★Methods

### Key resources table

**Table:** 

REAGENT or RESOURCE	SOURCE	IDENTIFIER
**Deposited data**		

Full dataset of tracked bees, including trajectories, calculated metrics, and comb maps	This paper	Zenodo: https://doi.org/10.5281/zenodo.6045860

**Experimental models: Organisms/strains**		

Honey bees	University of Konstanz apiary	N/A

**Software and algorithms**		

Beesbook tracking system	Wario et al. (2015); Boenisch et al. (2018)	Github: https://github.com/BioroboticsLab/bb_tracking
Analysis codes	This paper	Github: https://github.com/jacobdavidson/bees_lifetimetracking_2018data

### Resource availability

#### Lead contact

Information and requests for resources should be directed to and will be fulfilled by the Lead Contact, Michael L. Smith (mls0154@auburn.edu).

#### Materials availability

The study did not generate new unique reagents.

### Experimental model and subject details

Newborn worker bees were sourced from colonies headed by naturally mated queens from the University of Konstanz apiary. Individual age-matched cohorts were selected from eight different source colonies: cohorts A, H, M from colony c1; cohort D from colony c2; cohorts B, I, N from colony c3; cohort L from colony c4; cohorts C, E, K from colony c5; cohorts F, G from colony c6; cohort O from colony c7; cohorts J, P from colony c8.

### Method details

#### Observation hive and nest maps

This research was conducted at the University of Konstanz, Germany (47.6894N, 9.1869E). On 10 June 2018, the observation hive was installed with a single queen, 2,000 unmarked workers, and three frames of mixed brood and honey (”Deutsche-Normal” frames: 395 × 225 mm, observation hive: 490 × 742 mm; note that this observation hive is the largest, to date, to be used for automated tracking in honey bees (Wario et al., 2015; Boenisch et al., 2018; Wild et al., 2020; Bozek et al., 2021)). From 16 July to 3 Septempter 2018, every 4–6 days, we individually marked and introduced 200–600 newborn honey bees to the observation hive (total bees tagged: 5,343). Although tracking data was obtained continuously until 9 October 2018, we perform our analysis on a focus observation period of 16 July - 3 September, during which new cohorts were regularly introduced. Newborns were hatched overnight in an incubator kept at 34 ° C and 50 %RH, and marked the following morning with individual BeesBook tags (Wario et al., 2015; Boenisch et al., 2018). Tags are printed on paper and attached to the thorax of bees, and remain attached for their whole lives. From 16 July to 3 Sept 2018 (50 days) we recorded the observation hive at 3 frames per second using four Basler acA4112-20um cameras fitted with Kowa LM25XC lenses and the recording software Motif (Loopbio GmbH). The colony was illuminated with infrared light (850nm 3W LED’s), which is invisible to honey bees (Peitsch et al., 1992). The entire recording rig (observation hive, cameras, lighting) was kept in the dark, to mimic the natural conditions of the honeybee nest. Workers had free access to forage outside, through a entrance tunnel (2-cm diameter). To keep track of the colony’s weight, the observation hive was kept on a scale which logged its weight every hour (10g sensitivity, Wolf Waagen GmbH). To create a map of the nest, every 4–6 days we traced the contents of the observation hive onto plastic sheets by outlining the following: honey storage, pollen storage, brood, empty comb, wooden frames, peripheral galleries, and dances observed on the dance floor (as in (Smith et al., 2016); Figures 1B and S1). These plastic sheets were then scanned with an architectural scanner (Ruch-Medien, Konstanz), and digitized. By overlaying the bee trajectories upon the maps, we determined what type of nest environment an individual experienced (Figure 1B).

### Quantification and statistical analysis

#### Data processing and behavioral metrics

Using the BeesBook system (https://github.com/BioroboticsLab/pipeline), the raw image data were processed to detect and decode the individually marked bees (Boenisch et al., 2018; Wild et al., 2018, 2021). For each individual, its tag id, id detection confidence, position, and orientation were tracked over time, and stored in a PostgreSQL database. The death date of each marked individual was estimated using a Bayesian changepoint model (as in (Wild et al., 2021)). This method accounts for a low rate of erroneous detections in bees that have already died, and time periods when individuals are observed less frequently or not at all (e.g. while foraging). An individual’s death date was used as a cutoff for including data in subsequent calculations.

We chose metrics that represent space use within the nest (time on honey, brood, or dance floor, and exit distance), detection (time observed, time outside, number of outside trips, and number of dance floor visits), and movement/spatial localization (speed, circadian coefficient, dispersion, fraction of nest visited). Although some of these metrics are correlated (Figure S3A), they nonetheless represent different aspects of behavior, and we use the approach of combining multiple different metrics in order to obtain results that are robust to inclusion of specific metrics, as well as any particular parameter choices associated with each metric.

We processed the trajectory data to obtain the quantities used in the subsequent analyses by first averaging over 1-h time bins and saving the quantities of interest for each individual bee. The 1-h bins were used to speed up processing the large amount of data. All data points used in the analysis were above a detection confidence threshold of 0.8, and we calculated behavioral metrics for each bee that had a minimum of 10 detections in that hour. For time observed, number of outside trips, and number of dance floor visits, the per-day value is a sum across hours. The circadian coefficient is determined using the per-hour median speed over the coarse of a day. For the other 8 metrics, the per-day quantity is calculated as a weighted average across the hours in the day, where weightings are done according to the amount of time observed in that hour.

Table 1 shows a summary with definitions of all metrics used. Further details regarding calculations of substrate usage, circadian coefficient, and trips are described here.

Substrate usage is calculated using the comb substrate maps shown in Figure S1, grouping together capped and young brood into a single category. Note that dances were observed only within a limited time range (pink circles in Figure S1), but all occurred in a similar area. Defining the dance floor based on only direct observations would be overly restrictive, so we defined the dance floor area using a convex hull that contains all dances over the entire observation period (dashed pink line in Figure S1). Because the comb contents changed over time, and were not measured each day, we calculated substrate usage by a weighted average from values calculated using the substrate maps on the measurement days before and after the day in consideration. To illustrate this procedure, consider the day July 18, which has the closest measurement days of July 16 and 21. Denote the comb map from July 16 as A, and the comb map on July 21 as B. We first use the trajectory coordinates of the bee on July 18 to calculate two different approximate usage fractions: $S_{i}^{A}$, which is the fraction of time spent on substrate i as determined using map A, and $S_{i}^{B}$, which is the fraction of time spent on substrate i as determined using map B. The estimated substrate usage fraction for July 18 is calculated is calculated as a weighted average of these values:

$$f_{i}=w_{A}S_{i}^{A}+w_{B}S_{i}^{B},$$

where for this example the weights are $w_{A}=0.6$ and $w_{B}=0.4$, because the first comb measurement day is closer than the second to July 18. The nest comb contents over time were also determined by this same linear interpolation method between nest content measurement days.

The circadian coefficient is calculated as the correlation of median speed over the day with a daily rhythm that follows the sun. We approximate the daily rhythm with a sine curve of sin((h−m)/(2π)), where h is the hour of the day, and m is chosen so that the maximum of the curve coincides with the highest sun position of the day, which was approximately 13:30 CEST during the observation period. The circadian coefficient is then calculated as

$$C=\frac{\sum_{h=1}^{24}s_{h}\sin\left(\frac{h-m}{2\pi }\right)}{\sum_{h=1}^{24}s_{h}},$$

With this normalization the coefficient satisfies −1≤C≤1, where the extreme values only occur if the bee is not observed for the whole day. Positive values represent higher speed or only being observed during the day, while negative values represent higher speed or only being observed at night.

A bee’s barcode is not always detected when it is in the observation hive, for example if the bee is upside-down or in a dense crowd of other bees. Because of this, we used both detection and exit distance to estimate when a bee was outside. The time outside and number of outside trips are estimated by first calculating the time observed and median exit distance in 1-min bins over the coarse of a day. A bee is then estimated to have exited the nest in a time bin $t_{exit}$ if the time observed in $t_{exit}$ is less than a threshold of $t_{obs}$ = 2 s, and if the median exit distance in time bin $t_{exit}-1$ is less than a threshold $d_{exit}$ = 18.75 cm (1500 pixels). The bee is considered to have re-entered in bin $t_{enter}$ if the time observed in $t_{enter}$ is greater than or equal to $t_{obs}$. The values $t_{obs}$ and $d_{exit}$ are analysis parameters, and the results can depend strongly on the choice of $d_{exit}$; we choose the value of 18.75cm to represent a feasible median exit distance for a bee traveling to the exit during a 1-min period. With these results, we determine multiple instances of exit and re-entry times during the course of a day, and use this to calculate the number of outside trips (the number of times a bee is estimated to have exited the nest), as well as the time outside.

Note that dispersion and fraction of the nest visited are two complementary metrics which both represent how wide-ranging each bee is, regardless of where it tends to be located in the nest. While dispersion and fraction of the nest visited give similar results for continuous exploratory movement, they can yield different trends for other cases; for example, bursty movement can yield high fraction of nest visited yet low dispersion, while directed, straight-line back-and-forth movement can yield low fraction of the nest visited yet high dispersion.

#### PCA and clustering on single day metrics

Using the behavioral metrics (Table 1), we create a data matrix $M_{ij}$, where each row i represents one behavioral day, and columns j=1…12 are the different quantities. A behavioral day is only included if that bee was alive on the given day and had more than 1,000 detections over the whole day. This represents a total time observed of 5.5 min during a day; using this removes 7201 behavioral days with few detections (results are qualitatively similar whether these are included or not). In addition, we do not include bees on the first day they were introduced, because on this day there were not observed for a full 24 h. With this criteria i=1…53,032 behavioral days are included in the analysis. Although the total number of tagged bees was 5,343, the bees in cohorts A-F were tagged before filming began, and some died before 16 July. Due to this, and after filtering, we include data from a total of 4,193 unique bees in the analysis (the number is 4,229 before filtering for few detections).

Note that the nest contents – in particular the size of the honey and brood areas – change over time (Figure S1). We account for these changes in order to focus on variation among the activity of bees in the nest at a given time, instead of changes in substrate usage that result from a different nest composition. For honey and brood areas, we account for this by subtracting the nest content fraction from the individual bee substrate usage fraction for each day. The dance floor is unaffected, since it is defined as the same area over the course of the observation period.

Following standard procedures, we normalized the data matrix M so that the column mean is zero and the column standard deviation is 1. We then performed principal component analysis (PCA) on the resulting matrix to obtain the components shown in Figure 4A. The result of PCA is a matrix $U_{ij}$, where i represents behavioral days and j=1…12 for the PCA components (corresponding to the total number of behavioral metrics).

Next, we perform Ward hierarchical clustering, implemented in Python in the package scipy.cluster.hierarchy, to obtain the results shown in Figures 4 and S3.

Ward clustering minimizes the overall within-cluster variance. We write this as variance fraction: for n clusters, this is calculated as

(Equation 1) $$W\left(n\right)=\frac{1}{{\left|\left|\text{M}\right|\right|}^{2}}\sum_{q=1}^{n}\sum_{i\in q}\sum_{k}{\left(M_{ik}-{\left\langle M_{jk}\right\rangle }_{j\in q}\right)}^{2},$$

where ${\left\langle \cdot \right\rangle }_{j\in q}$ represents an average over the indices j that are elements of cluster q, and $\|M{\|}^{2}=\sum_{i,j}M_{ij}^{2}$ is the squared magnitude of the data matrix. This is shown in Figure S3.

We use t-SNE embedding (Maaten and Hinton, 2008) implemented in openTSNE (Poličar et al., 2019), with parameters of perplexity = 30 and n_iter = 1000, and initial conditions set by the first two PCA dimensions to obtain the behavioral day embeddings shown in Figure 4. This package enables the mapping of new data to existing embeddings, which we used to show the average bee-life trajectories on top of the behavioral day embedding (Figure 5E).

#### Variance fraction explained

To compute the fraction of the total variance explained by age, cohort, or a combination of factors, we use the same procedure as in Equation 1, but instead generalize to use some grouping {G} instead of a certain number of clusters. The grouping {G} can be defined to include bees of a certain age, bees of a certain cohort, or both of these (bees of a certain cohort having a certain age). The variance fraction for all metrics is then calculated as

(Equation 2) $$W\left(\left\{G\right\}\right)=\frac{1}{{\left|\left|M\right|\right|}^{2}}\sum_{q\in \left\{G\right\}}\sum_{i\in q}\sum_{k}{\left(M_{ik}-{\left\langle M_{jk}\right\rangle }_{j\in q}\right)}^{2}.$$

For a certain metric k, this is simply

(Equation 3) $$W\left(\left\{G\right\},k\right)=\frac{1}{\sum_{i}M_{ik}^{2}}\sum_{q\in \left\{G\right\}}\sum_{i\in q}{\left(M_{ik}-{\left\langle M_{jk}\right\rangle }_{j\in q}\right)}^{2}.$$

#### Bee-life

We use the behavioral metrics (Figure 3) computed over multiple days in order to compare the lifetime behavioral trajectories of individual bees. The results in Figure 4 treat each day for each bee separately, and each row of M represents one behavioral day. Building on this notation, we know that a bee’s life is made up of multiple behavioral days. To ask about bee-lives with similar patterns and changes of activity as a bee ages, we filter and transform the data, and perform PCA on the behavioral metrics of each bee over time.

Individual bees have different lifespans; because of this, we did not include all bees in the lifetime analysis, but only those that were observed for at least 10 days. To compare lives we also need to a set a maximum value of the number of days to compare; we use a maximum of 25 days as value that is representative of the lifetime behavioral changes of bees. Since PCA cannot be performed if values are missing (which occurs, for example, after a bee has died), we use per-age average values of each metric to fill in missing values of the behavioral metrics for the purposes of PCA and clustering.

The tensor $B_{\alpha tj}$ is used to represent bee-lives, where α is for individual bees, t is an index over the days in the bee’s life, which goes from 0 to $l_{\alpha }$, where $l_{\alpha }$ is the total number of days the bee lived, and j=1…12 is an index over the component values of M. To analyze how different one bee’s life is from another’s, we must consider that all bees did not live for the same number of days. Because of this, we use a parameter $A_{max}=25$ for the maximum age used in the bee-life analysis. Because some bees did not live a total of $A_{max}$ days, and even if a bee was alive there could be some days where it was not detected by the tracking system, we only include bees for the lifetime analysis that had $D_{min}=10$ days or more in the behavioral day data matrix. With these criteria, and also only keeping bees from cohort G onward, i.e. bees with birthdates within the observation period, we include 2027 bees in the bee-life analysis. We note that $A_{max}$ and $D_{min}$ are analysis parameters and quantitatively affect results, although we found that different values of these parameters lead to qualitatively similar interpretations in the differences among bee-lives. We used averages to fill in values of the bee-life matrix for the purposes of PCA and clustering, because PCA cannot be calculated on a matrix that has missing values. Let $h_{tj}={\left\langle B_{\alpha tj}\right\rangle }_{\alpha }$, where the notation ${\left\langle \cdot \right\rangle }_{\alpha }$ represents an average over the index α, denote the average behavioral metrics for each day of the lives of bees that were observed. For a bee that was dead or not observed on day t of its life, we fill these values by setting ${B_{\alpha tj}|}_{\text{bee}\;\alpha \;\text{dead or not observed on day}\;t}=h_{tj}$. We use $h_{tj}$ instead of zeros to fill values for the bee-life distance metric, because although the column average of M is zero, the average conditional on the age of the bee is nonzero, and therefore filling with zeros would bias the results. After this filtering and processing, we use the bee-life matrix $B_{\alpha tj}$ as input to PCA and clustering, to obtain the results shown in Figure 5.

We obtain that PCA 1 explains 11.4% of the total variance, PCA 2 explains 8.4% of the total variance, and further components explain a smaller fraction of the total variance (Figures 5 and S4). Note that because the input is high-dimensional, with $A_{max}\times 12=300$ columns, the fraction of the variance explained by any single mode is relatively small, with an average at 0.31%, and therefore the first two modes represent strong patterns in the data because they are very high above this average variance fraction.

We use $B_{\alpha tj}$ as input to Ward hierarchical clustering obtain the clusters shown in Figure 5, and use a 5 cluster grouping to highlight differences along the first two dominant life PCA modes (Figure S4).

## Data Availability

• The data and code needed to reproduce the results in this publication are available at Github: github.com/jacobdavidson/bees_lifetimetracking_2018data. • The full dataset associated with tracking bees during summer 2018 is is publicly availableat Zenodo**:** https://doi.org/10.5281/zenodo.6045860 and is also listed in the Key resources table. This full dataset includes the x-y trajectories, behavioral metrics calculated at different time intervals (1-h, 5 min, and 1 min intervals), and comb maps. • Any additional information required to reanalyze the data reported in this paper is available from the lead contact upon request.
