# Supplementary material for: Behavioral variation across the days and lives of honey bees
Source: iScience. 2022 Aug 8;25(9):104842. doi: 10.1016/j.isci.2022.104842 (PMC9418442; doi:10.1016/j.isci.2022.104842)
Supplement: Document S1. Figures S1–S5 [file mmc1.pdf]

iScience, Volume *25*

Supplemental information

Behavioral variation across the days and lives of honey bees

Michael L. Smith, Jacob D. Davidson, Benjamin Wild, David M. Dormagen, Tim Landgraf, and Iain D. Couzin

Supplemental figures for *Behavioral variation across the days and lives of honey bees*

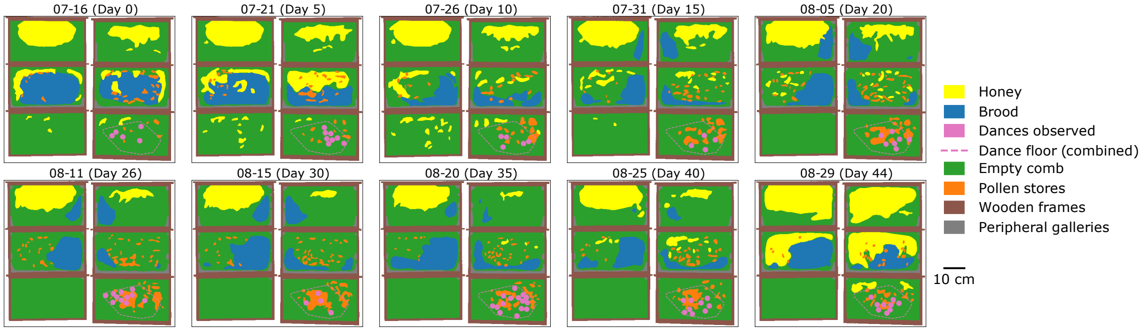

Figure S1: **Comb contents over the observation period.** Figure shows the nest contents and tracings. The pink dashed line in each shows the "combined dance floor", which is defined as a convex hull that contains the locations of all observed dances. The relative locations of the nest contents remained stable throughout the experiment, with honey stored at the top of the nest, brood reared in the center, and a dance floor at the bottom of the nest, near the nest entrance.

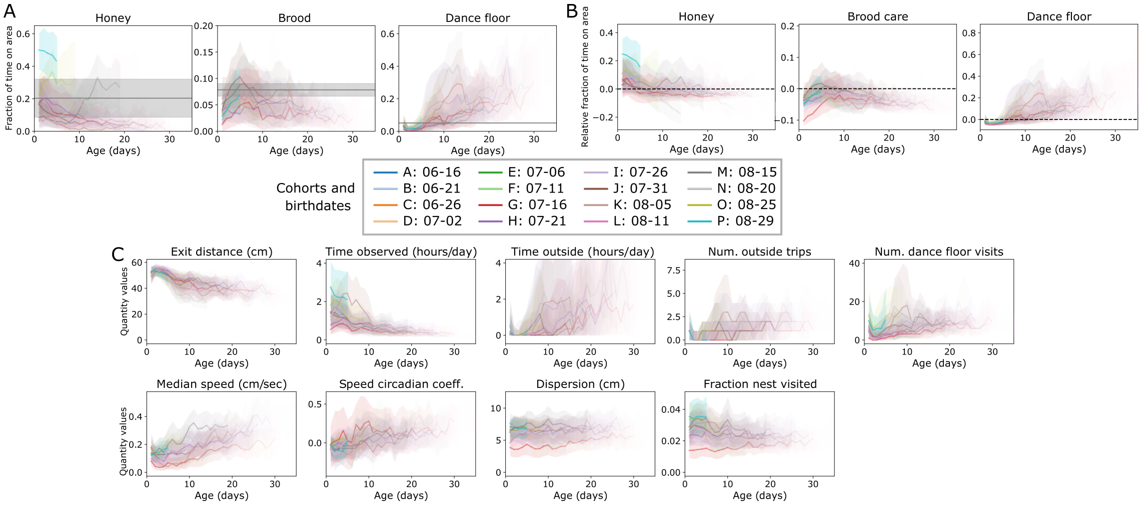

Figure S2: **Substrate and other quantities with age.** See also Figure 3. Cohorts are indicated by the different colors for each plot. (A) Fraction of time spent on honey and brood. The gray line and shaded area shows the mean and standard deviation of the amount of honey or brood in the nest over time. (B) Fraction of time on honey and brood relative to nest contents, determined by subtracting the average contents of the nest for a given day. (C) Other behavioral metrics with age.

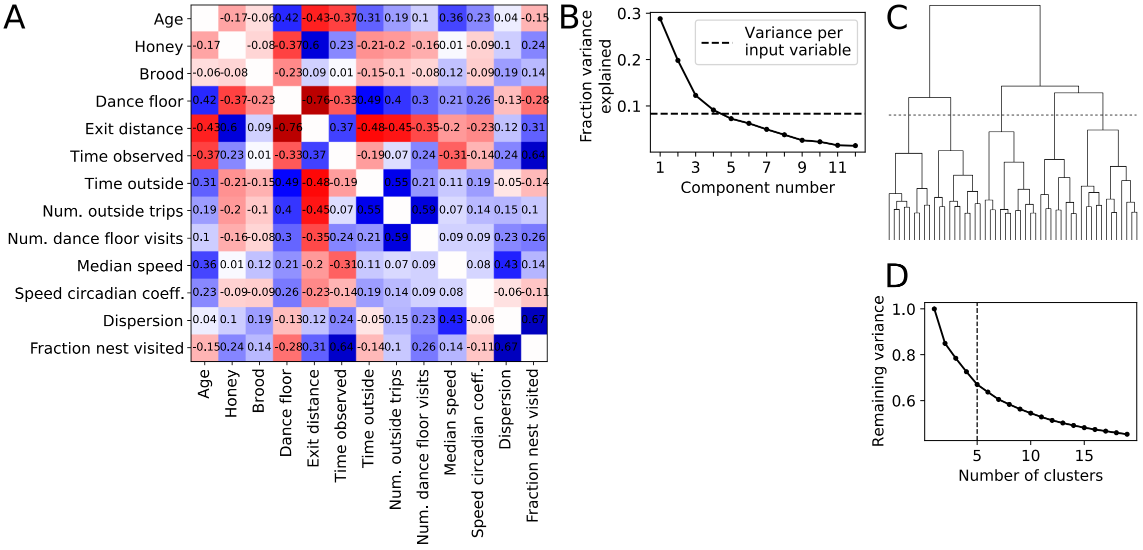

Figure S3: **Correlation between single-day quantity values, PCA variance explained, and behavioral day clustering results.** (A) Pearson (ranked) correlation coefficient shown, for all behavioral metrics as well as age. Blue indicates a positive correlation, and red indicates a negative correlation; the values of the correlation coefficient are shown for each pair of quantities. (B) Variance explained per behavioral day PCA component. Dashed line shows the variance per input column of M_{ij}, i.e. the contribution of each behavioral metric to the total variance. (B) Clustering dendogram and (D) average remaining variance as a function of the number of clusters (Eq. 1). In both, the dashed line shows the distance cut-off for a 5-cluster division.

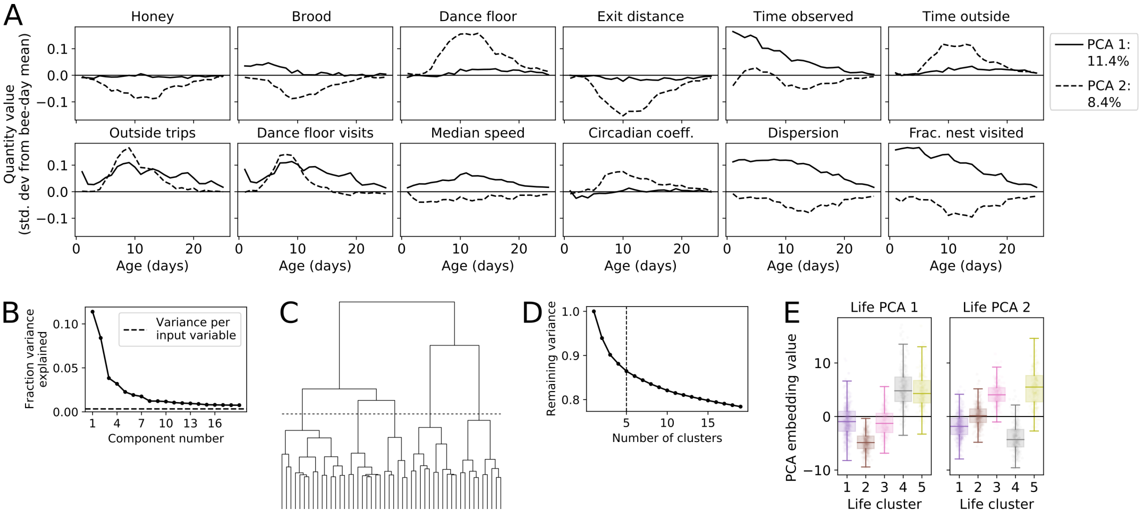

Figure S4: **Lifetime PCA decomposition and clustering.** (A) The first two life-PCA modes plotted in terms of behavioral metrics with age, using normalized quantities with the same units as Figure 4 (i.e. zero represents the mean of a certain behavioral metric across all behavioral days). (B) Variance explained per bee-life PCA component. Dashed line shows the variance per input column of $B_{\alpha t j}$, i.e. the contribution of each behavioral metric j for a bee with age t days to the total variance. (C) Clustering dendogram and (D) average remaining variance as a function of the number of clusters. In both, the dashed line shows the distance cut-off for a 5-cluster division. (E) Life-PCA embedding values, obtained by projecting lifetime behavioral metrics onto the lifetime PCA decomposition shown in A and Figure 5A.

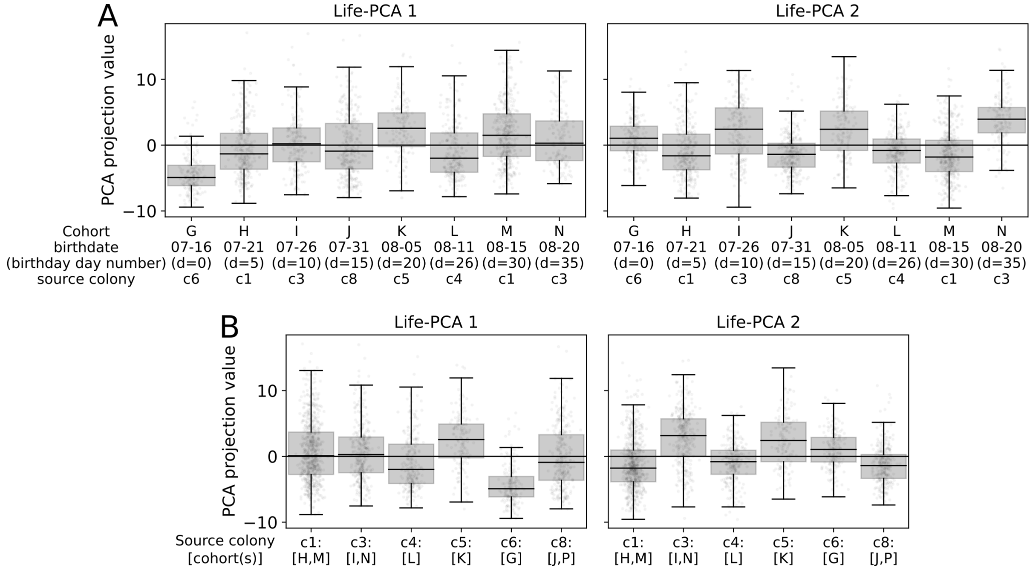

Figure S5: **Cohort and source colony distributions of lifetime behavior.** The distribution of life-PCA embeddings for bees that were included in the lifetime analysis. Note that only cohorts from G onward are included in the lifetime analysis, because these bees have birthdates within the observation period. The life-PCA modes 1 and 2 are shown in Figure 5. (A) Per-cohort distributions of lifetime PCA 1 and 2 projections. Cohorts are sorted chronologically, with birthdate and associated source colony shown in the label. (B) Per-source colony distributions of lifetime PCA 1 and 2. Associated cohorts are listed in the label.
